# A Green Synthesis Route to Derive Carbon Quantum Dots for Bioimaging Cancer Cells

**DOI:** 10.3390/nano13142103

**Published:** 2023-07-19

**Authors:** Karthiga Anpalagan, Jimsheena Valiyakath Karakkat, Raz Jelinek, Nila Nandha Kadamannil, Tian Zhang, Ivan Cole, Kulmira Nurgali, Hong Yin, Daniel T. H. Lai

**Affiliations:** 1 Institute of Health and Sport (IHeS), Victoria University, Melbourne, VIC 3011, Australia; karthiga.anpalagan@live.vu.edu.au (K.A.); jimsheenavk@gmail.com (J.V.K.); kulmira.nurgali@vu.edu.au (K.N.); 2 Department of Chemistry and Ilse Katz Institute for Nanoscale Science and Technology, Ben Gurion University of the Negev, Beer Sheva 8410501, Israel; razj@bgu.ac.il (R.J.); kadamann@post.bgu.ac.il (N.N.K.); 3 Department of Chemical and Biological Engineering, Monash University, Melbourne, VIC 3800, Australia; tian.zhang1@monash.edu; 4 School of Engineering, RMIT University, Melbourne, VIC 3000, Australia; ivan.cole@rmit.edu.au

**Keywords:** carbon quantum dots, hydrothermal method, toasting, facile synthesis, bioimaging, cancer cells

## Abstract

Carbon quantum dots (CQDs) are known for their biocompatibility and versatile applications in the biomedical sector. These CQDs retain high solubility, robust chemical inertness, facile modification, and good resistance to photobleaching, which makes them ideal for cell bioimaging. Many fabrication processes produce CQDs, but most require expensive equipment, toxic chemicals, and a long processing time. This study developed a facile and rapid toasting method to prepare CQDs using various slices of bread as precursors without any additional chemicals. This fast and cost-effective toasting method could produce CQDs within 2 h, compared with the 10 h process in the commonly used hydrothermal method. The CQDs derived from the toasting method could be used to bioimage two types of colon cancer cells, namely, CT-26 and HT-29, derived from mice and humans, respectively. Significantly, these CQDs from the rapid toasting method produced equally bright images as CQDs derived from the hydrothermal method.

## 1. Introduction

Carbon quantum dots (CQDs), as an emerging family of fluorescent nanomaterials, have been a focal point for worldwide researchers over the past decade because of their unique properties, such as tunable photoluminescence, facile fabrication techniques, biocompatibility, low toxicity, and good photostability [1,2,3,4,5]. These nanoclusters, with an average diameter of less than 10 nm, were first discovered by Xu et al. In 2004 [6], Sun et al. [7] were the first to produce and designate these fluorescent particles as CQDs. Their fluorescence property has found applications in various optoelectronic devices [8,9,10] and created opportunities for bioimaging, biosensing, and disease diagnosis [11,12,13,14,15,16,17]. Because of the cost-effectiveness and energy efficiency of the fabrication techniques, together with the low toxicity of the CQDs, they can potentially replace industrial fluorescent dyes used in the medical sector for real-time bioimaging. CQDs have been considered as possible diagnostic and therapy tools for cancer detection and bioimaging owing to their excellent performances in precisely targeted bioimaging [18,19,20]. Fluorescent CQDs also demonstrate sensing ability towards a broad range of analytes, such as cations, anions, small molecules, macromolecules, cells, and bacteria [21,22,23,24].

Typically, two techniques are commonly used in the fabrication of CQDs: “top-down” and “bottom-up” methods. The former involves breaking down carbonaceous materials via chemical or physical approaches. The latter requires the carbonization of small organic molecules or step-by-step chemical fusion of small aromatic molecules [25,26,27]. Nowadays, there has been much interest in exploring and improving bottom-up approaches because of the ease of operation, precise control of precursor molecules, low cost, and environmental friendliness [28,29]. Among all of the bottom-up routes, two major techniques, namely, hydrothermal/solvothermal and pyrolysis, are used for fabricating CQDs. The hydrothermal method is a popular fabrication route that adds value to carbohydrates through an environmentally friendly method [30]. This technique is typically performed at 180 to 260 °C for 4 to 12 h under water saturation pressure [31]. Under these conditions, water is more reactive and behaves as a nonpolar solvent because of the high ionic product and low dielectric constant [32]. An autoclave reactor with Teflon-lined vessels is generally used to perform this fabrication under specific conditions. On the other hand, thermal decomposition/thermolysis is a technique that carbonizes carbohydrates using excess heat under atmospheric conditions. This process takes place above 200 degrees for a few minutes. The carbonization degree plays a major part in this method because incomplete carbonization produces luminescent carbon-based quantum dots, and complete carbonization results in carbon residues with weak emission [33]. However, it is a facile and cost-effective route that offers a quicker fabrication suitable for industrial scale-up. 

The need for biocompatibility and cost effectiveness leads to the fabrication of CQDs from food resources. A large selection of vegetables, fruits, and plant extracts, such as rice [34], carrot [35], cherry tomato [36], lemon peel [37], onion waste [38], taro peel [39], watermelon peel [40], and banana peel [13], have been reported. Generally, synthetic chemicals are used in fabrication processes, and they curtail the biocompatibility of the CQDs derived from food resources. Bread is an edible carbohydrate resource previously used to produce fluorescent CQDs. However additional chemicals, such as methanol [41] and nitric acid [42], have to be added during the synthesis. Very recently, Eswaran et al. reported highly fluorescent CQDs prepared from bread waste without any additional chemicals. The hydrothermal synthesis involved the high consumption of energy and a long reaction time (13 h) [43]. We previously demonstrated that biocompatible CQDs could be fabricated from bread through a chemical-free process [44]. However, the fluorescence of these CQDs is unsatisfactory, and their quantum yields were not reported. 

The aim of this study was to discover a fast, cost-effective, and energy-efficient process to fabricate biocompatible and highly fluorescent CQDs for bioimaging. Herein, three types of bread (white bread, whole meal bread, and multigrain bread) were used as precursors without any additional chemicals. The toasting method and hydrothermal method were compared at their optimal conditions to evaluate their efficiency. The tailored CQDs were studied in terms of their structure, composition, photoluminescence, cytotoxicity, cellular internalization, and bioimaging capability. 

## 2. Materials and Methods

### 2.1. Fabrication of CQD Samples

Two different synthesis routes were investigated on three types of bread, namely, white bread, whole meal bread, and multigrain bread, providing six different samples. These packs of sliced bread were purchased from a local supermarket. For the toasting method, two slices of each type of bread were separately toasted using a household bread toaster (TARSST19B, Target Corporation, Williams Landing, Australia) at the maximum heat setting for 5 min. The heating temperature was increased from room temperature to 240 °C during this toasting period. The temperature was measured using an infrared temperature probe (Sovarcate, Guangzhou, China) every minute, and the heating rate was calculated. This experimental condition was optimized based on the fluorescence emission of the resultant CQDs. The carbon residues from the charred bread slices were carefully scrubbed out and grounded using a mortar and pestle. The grounded fine powder from each type of bread (3 g ± 0.1 mg) was dispersed in 30 mL of Milli-Q water and sonicated (Soniclean, LABOUIP Technologies, Bayswater, Australia) for 5 min. The mixtures were then centrifuged (MSE centrifuge, Thomas Scientific, Swedesboro, NJ, USA) for 5 min at 3000 rpm. The resulting supernatants were filtered using a number 1 (90 mm) filter paper (Whatman, GE Healthcare UK Limited, Amersham, UK). A sterile syringe filter unit (Minisart, Sartorius, Gottingen, Germany) of 0.2 µm was used to purify the sample and prevent bacterial growth [45]. The sample from white bread, whole meal bread, and multigrain bread were labeled as CQD T1, CQD T2, and CQD T3, respectively. All samples were freeze-dried and stored at −20 °C for further analysis.

The second method used to fabricate CQDs was hydrothermal synthesis. Each type of bread (3 g ± 0.1 mg) was separately dispersed in 30 mL of Milli-Q water by sonication (Soniclean, LABOUIP Technologies, Bayswater, Australia) for 5 min and transferred into three different 50 mL autoclave chambers (Robotdigg equip makers, Hong Kong, China) and heated at 180 °C for 4 h in an oven (RHTOV2HP, Russell Hobbs, Spectrum brands, Braeside, Victoria, Australia). This fabrication process was optimized based on the fluorescence emission of the resultant CQDs. After the heating, the solutions were cooled to room temperature naturally. Then, the mixtures were centrifuged, filtered, and purified as previously described. The samples from the white bread, whole meal bread, and multigrain bread were labeled as CQD H1, CQD H2, and CQD H3, respectively. These samples were also freeze-dried and stored at −20 °C for further analysis.

### 2.2. Characterization of CQD Samples

#### 2.2.1. Transmission Electron Microscopy (TEM)

A 0.01 wt.% CQD solution was prepared, and 4 µL of the solution was placed on a carbon-coated copper grid (Carbon Type B, 400 mesh, with Formvar (Ted Pella, Inc., Redding, CA, USA)). The solutions were allowed to evaporate under ambient conditions. The images were collected at varied magnifications using a Tecnai T12 G2 TWIN microscope (FEI company, Hillsboro, OR, USA) operating at 120 kV.

#### 2.2.2. Fluorescence Measurements

Fluorescence measurements were carried out on a Spectro fluorophotometer RF—5301PC (Shimadzu Europe, Duisburg, Germany) with a 1 mm PMMA cuvette (Shimadzu, Duisburg, Germany). The emission spectra were recorded at excitation wavelengths from 360 nm to 440 nm in 20 nm increments. 

#### 2.2.3. Fourier-Transform Infrared Spectroscopy (FTIR)

The FTIR spectra of the lyophilized samples were determined using an FTIR spectrometer (Perkin Elmer, Waltham, MA, USA). An average of 16 scans with a resolution of 4 cm^−1^ was performed within the range of 4000–400 cm^−1^.

#### 2.2.4. Quantum Yield Measurement

The fluorescence quantum yield was obtained on a Pico Quant FT300 using an integrated sphere. The quartz cuvette (1 cm × 1 cm × 5 cm) containing the sample solution was positioned in the “IN mode” with 20-degree tiling, where the 423 nm excitation laser was directly transmitted through the cuvette. For the measurement of low quantum yield values, an emission attenuator (attenuation level at 100) was applied for less than 445 nm, which was counted in the quantum yield calculation. 

### 2.3. Cell Culture

The synthesized CQDs were tested on the human colon adenocarcinoma cell line, HT29, and mouse colon carcinoma cell line, CT-26. Briefly, the cells were maintained in DMEM medium supplemented with 10% heat-inactivated fetal bovine serum and penicillin/streptomycin (100 units/mL of penicillin and 100 μg/mL of streptomycin) in a humidified incubator with 5% CO_2_ atmosphere at 37 °C. The culture medium was changed every 2 to 3 days, and cells were harvested for experiments at the logarithmic phase using TrypLE™ Select (Gibco™) (Thermo Fisher Scientific, Scoresby, Australia) and diluted with the complete media. 

Square glass coverslips (22 mm × 22 mm) were coated with poly-l-Lysine and placed in 6-well tissue culture plates, and 5 × 10^5^ cells were seeded to each well in complete media. After 24 h of incubation, the CQDs were dissolved in phosphate buffer saline (PBS), filtered through 0.2 µM filters, and added to the wells at a final concentration of 1 mg/mL. Sterile PBS at an equal volume was used in the control wells. After 24 h of incubation with the CQDs, the coverslips were fixed with 10% formalin for 15 min, followed by washes in PBS. The washed coverslips were flipped and mounted onto glass slides using ProLong™ Gold Antifade Mountant (Thermo Fisher Scientific, Scoresby, Australia) with or without the nuclear counterstain 4,6-diamidino-2-phenylindole (DAPI) (Invitrogen P10144, P36931) to a final concentration of 300 nM. The cells were visualized with a Leica Stellaris 5 confocal microscope at 20× or 63× magnifications. All images were converted to the tagged information file format and processed with Microsoft PowerPoint. 

### 2.4. In Vitro Cytotoxicity Evaluation

The cytotoxicity of the CQDs was evaluated with a 3-(4,5-dimethylthiazol-2-yl)-2,5-diphenyltetrazolium bromide (MTT) assay. Briefly, approximately 5000 cells were seeded in each well of a 96-well plate. After 24 h of incubation, varying concentrations (0–1.5 mg/mL) of each CQD were added and incubated for another 24 h. The cells were washed with PBS, and 10 µL of freshly prepared MTT (0.5 mg/mL) solution was added to each well. After 4 h, 100 µL of DMSO was added to dissolve the formazan crystals that had formed. The plate was read at 565 nm on a microplate reader, and the data were recorded. 

## 3. Results

### 3.1. Characterization of CQDs

#### 3.1.1. Transmission Electron Microscopy

**Figure 1:**
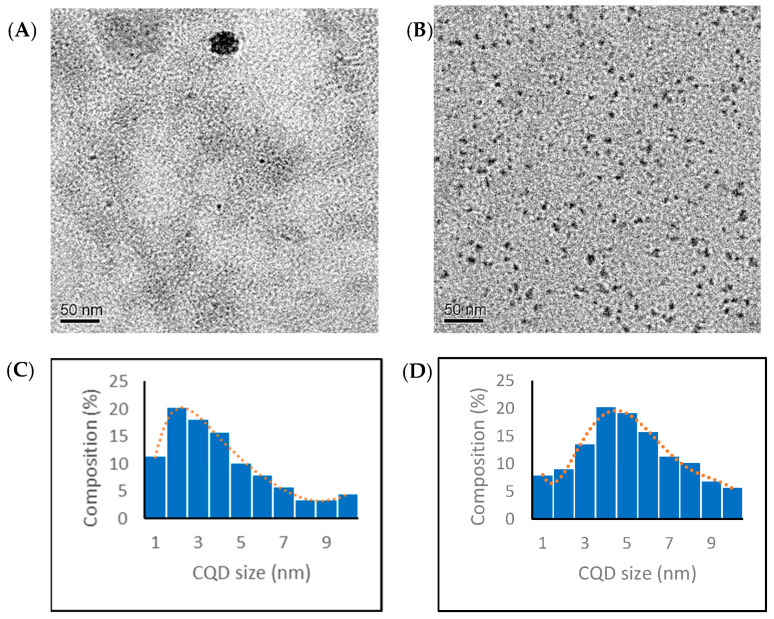
TEM images and size distribution histogram of (**A**,**C**) CQD T1 and (**B**,**D**) CQD H1, respectively. The width of a blue bar corresponds to the lower or upper limit of the size range and the height of the bar corresponds to the percentage of particles within that size range. The red dotted line shows the size distribution curve.

The CQDs prepared by the toasting and hydrothermal methods were observed with TEM. All of the CQDs exhibited spherical shapes with diameters less than 10 nm. Figure 1 shows a typical TEM image of the CQD T1 and CQD H1 and their size distribution histograms. The histogram of the CQD T1 (toasting) is left-skewed with smaller particles (2 nm to 4 nm), and the histogram of the CQD H1 (hydrothermal) is bell-shaped, with particle sizes mostly between 3 nm and 7 nm. From the size distribution histograms, the average particle sizes of the CQD T1 and CQD H1 were determined as 4.0 ± 2.4 nm and 5.2 ± 2.2 nm, respectively. A t-test suggested a p-value less than 0.001, i.e., the sizes from the toasting and hydrothermal methods were significantly different. The hydrodynamic diameters of the CQD T1 and CQD H1 were 3.5 ± 0.9 and 6.2 ± 1.7 nm obtained with dynamic light scattering (DLS), consistent with the particle size measured using TEM. 

#### 3.1.2. Optical Characterization

**Figure 2:**
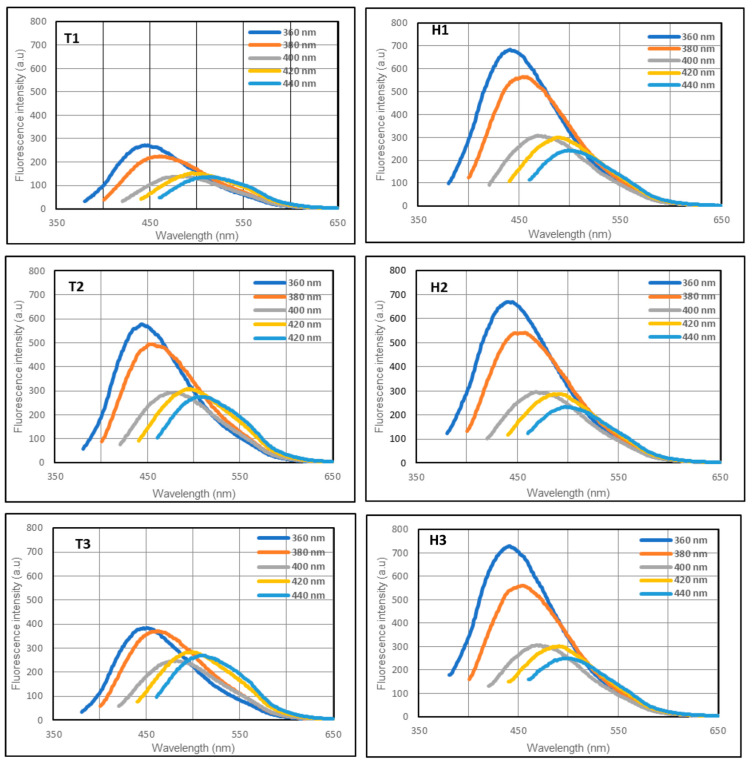
Emission spectra of the samples excited by light at 360, 380, 400, and 420 nm. T1 (white), T2 (whole meal), and T3 (multigrain) are samples from the toasting method, and H1 (white), H2 (whole meal), and H3 (multigrain) are samples from the hydrothermal method.

One of the remarkable features of CQDs is the strong broadband photoluminescence of their colloidal solutions. The photoluminescence spectra of all six samples were obtained using five excitation wavelengths from 360 nm to 440 nm (Figure 2).

The samples from both methods were fluorescent over a wide range of excitation wavelengths. For the hydrothermal method, all samples were brighter, and there was only a minimal variation in the photoluminescence among the three types of bread. For the samples derived from the toasting method, the emission of T1 is slightly lower than those of T2 and T3. The whole meal bread derived CQDs exhibited better fluorescence in both fabrication methods compared to the CQDs derived from white bread and multigrain bread. The overall photoluminescence trend of the samples was similar, with a peak emission at 360 nm excitation wavelength.

#### 3.1.3. Quantum Yield Measurement

As shown in Table 1, the samples derived from the toasting method (CQD T1, CQD T2, and CQD T3) exhibited a lower quantum yield compared to those from the hydrothermal method (CQD H1, CQD H2, and CQD H3). However, the difference was not significant in the bioimaging results, which will be discussed later. The CQD T2 and CQD H2 fabricated from whole meal bread possessed a higher quantum yield than the CQDs derived from the other two types of precursors.

#### 3.1.4. FTIR Characterization

**Figure 3:**
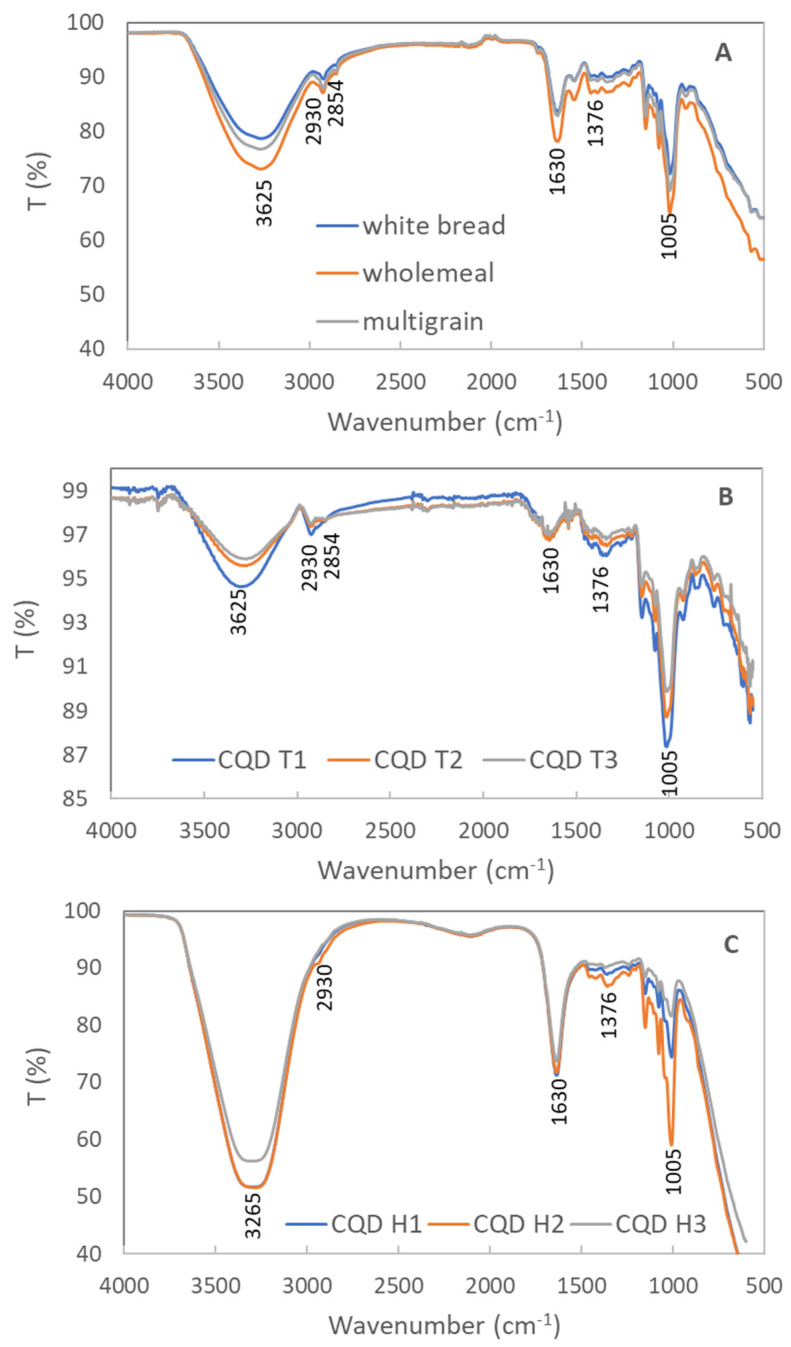
FTIR spectra of (**A**) precursors of samples (white bread, whole meal bread, and multigrain bread); (**B**) samples from the toasting method (CQD T1-white bread, CQD T2-whole meal bread, and CQD T3-multigrain bread); (**C**) samples from the hydrothermal method (CQD H1-white bread, CQD H2-whole meal bread, and CQD H3-multigrain bread).

In the spectra of the bread precursors, the broad band at 3265 cm^−1^ and the strong peak at 1630 cm^−1^ were attributed to the stretching and bending vibrations of the hydroxyl group (-OH), respectively [46]. The peaks centered at 2930 and 2854 were assigned to the stretching vibrations of the C-H bonds, and the peak at 1376 cm^−1^ was the bending vibration of the C-H bonds [47]. The strong peak at approximately 1005 cm^−1^ corresponded to the C-O-C group in the carbohydrates. The FTIR spectra of all of the CQDs showed a significant peak at 1005 cm^−1^, suggesting that the C-O-C bonds were retained in the prepared CQDs. The FTIR spectra of the samples prepared from the same method were similar. Comparing the spectra of the samples prepared using the different methods, some differences were observed. The C-H peaks (2930, 2854, and 1376 cm^−1^) were more distinct in Figure 3B than in Figure 3C, implying that the toasted samples had more hydrocarbon bonds. The hydrothermal samples had stronger -OH peaks (3265 and 1630 cm^−1^) than the toasted samples, confirming that water was involved in the hydrothermal process and introduced more -OH functional groups in the CQDs.

### 3.2. Cytotoxicity of CQDs

**Figure 4:**
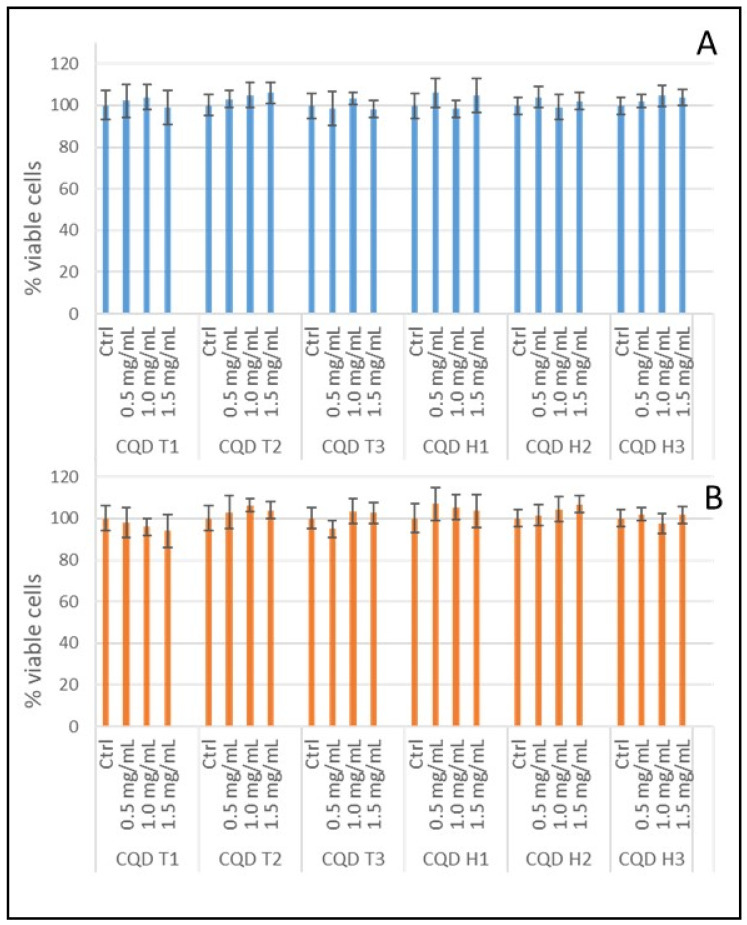
Cell viabilities of CQDs: (**A**) MTT assay of CT-26 cells with CQD T1, CQD T2, CQD T3, CQD H1, CQD H2, and CQD H3; (**B**) MTT assay of HT-29 cells with CQD T1, CQD T2, CQD T3, CQD H1, CQD H2, and CQD H3. All CQDs were tested at 0.5, 1.0, and 1.5 mg/mL concentrations.

Cytotoxicity is one of the major drawbacks of several fluorescent dyes used for bioimaging, which limits the long-term studies of cells. The cytotoxicity of our synthesized CQDs was evaluated using a standard MTT cell viability assay. Both CT-26 and HT-29 cells were incubated with 0–1.5 mg/mL of as-synthesized CQDs for 24 h. More than 95% of the cells survived even at the highest concentration tested, suggesting our CQDs had low cytotoxicity and, hence, excellent biocompatibility (Figure 4). 

### 3.3. Bioimaging Application

Imaging cells, tissues, organs, and other biological entities are vital for biomedical research. Organic dyes and inorganic semiconductor-based fluorophores are widely used for cellular imaging and bioimaging applications. Fluorescence imaging has harnessed particular interest with its real-time capability and high sensitivity. Nevertheless, photobleaching, poor water solubility, and toxicity are some of the obstacles to using conventional dyes in biological applications [48]. The six CQDs described in this study were synthesized from natural food sources via a green route. They exhibited tunable fluorescence, ultra-small size, photostability, and water solubility, making them promising candidates for bioimaging [49,50]. 

All CQDs were tested in two different types of colon cancer cell lines, namely, CT-26 and HT-29, derived from mice and humans, respectively. The cells were grown on glass coverslips for 24 h, followed by the addition of CQDs to a final concentration of 1 mg/mL culture medium. The cells are then incubated for 24 h, after which they were fixed in 10% formalin and imaged using confocal microscopy. 

**Figure 5:**
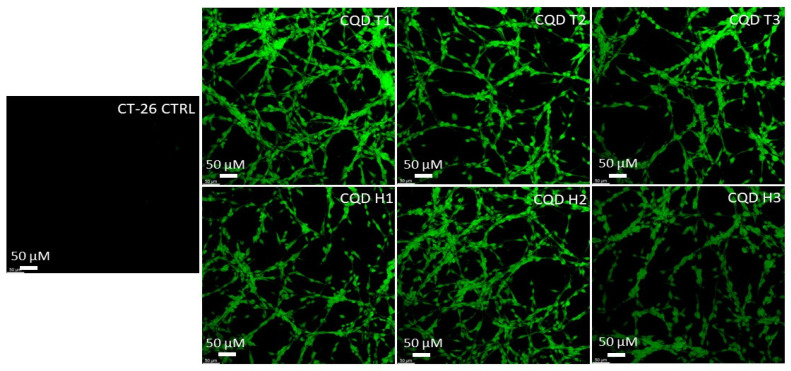
Confocal microscopy images of mouse colon cancer cells (CT-26) incubated with CQDs for 24 h. All images were obtained through the green channel (excitation peak at 431 nm and an emission peak at 540 nm) at 20× magnification.

**Figure 6:**
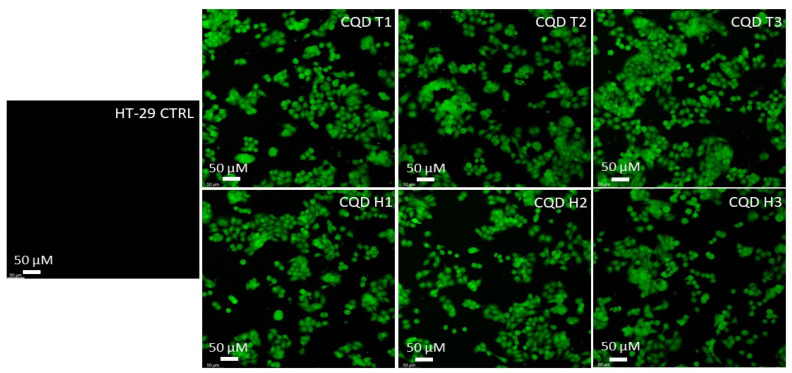
Confocal microscopy images of human colon cancer cells (HT-29) incubated with CQDs for 24 h. All images were obtained through a green (excitation peak at 431 nm and an emission peak at 540 nm) channel at 20× magnification.

The results show that CQDs can cross the cell membranes of both mice (CT-26) and humans (HT-29) colon carcinoma cells. Figure 5 and Figure 6 show that the cells were lit brightly by the internalized CQDs with green fluorescence, and the CQDs derived by both methods showed comparable fluorescence intensity.

**Figure 7:**
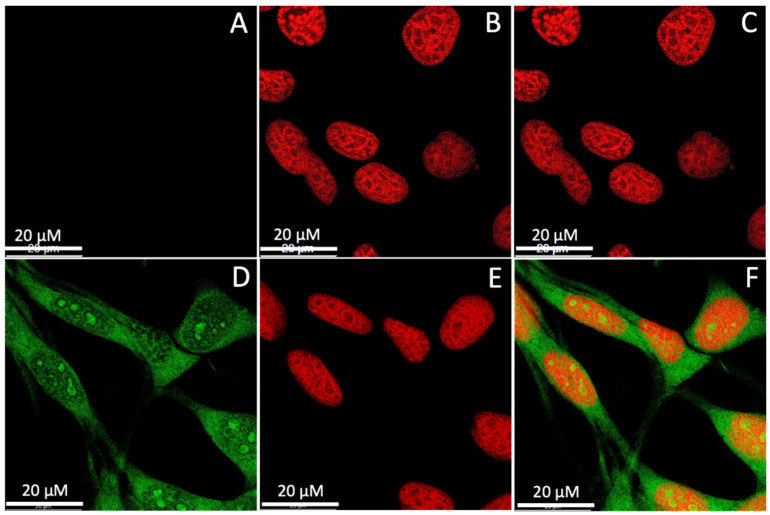
Confocal microscopy images of CT26 cells at a 63× magnification: (**A**–**C**) control cells only stained with propidium iodide not CQD H2; (**D**–**F**) cells incubated with CQD H2 and stained with propidium iodide. (**A**,**D**) Imaged through the green channel; (**B**,**E**) imaged through the red channel; (**C**,**F**) overlay of images from the green and red channels.

The localization of the CQDs inside a cell was investigated to evaluate the efficacy of using the CQDs for imaging applications. The CT-26 cells were incubated with CQD H2 for 24 h, fixed, and counterstained with the nuclear stain propidium iodide. Cells without CQDs but in the presence of propidium iodide were used as control (Figure 7A–C). In Figure 7A, the cells without CQDs did not show any signal from the green channel. In Figure 7B, only the nuclei were stained by propidium iodide (red) in the absence of CQDs without any visible cytoplasmic staining. As shown in Figure 7D, cytoplasm with green fluorescence derived from CQDs can be clearly observed, demonstrating the successful internalization of CQDs into the cells. Because of their ultra-small size, CQDs are also visible in the nuclear region, albeit on a smaller scale compared to the cytoplasmic localization. In Figure 7F, an overlay of images from the green and red channels clearly differentiates the cell cytoplasm and nucleus. 

## 4. Discussion

### 4.1. Fabrication of CQDs

The toasting and hydrothermal methods produced fluorescent CQDs for bioimaging. Their average energy consumptions are compared in Table 2. The energy calculation was based on the power output multiplied by the time. The hydrothermal method consumed almost five times the amount of energy compared to the toasting method. 

### 4.2. Characterization of CQDs

Both fabrication techniques yielded fluorescent CQDs using chemical-free processes. Almost all of the fabricated CQDs were less than 10 nm in diameter and agreed with previously published reports [51,52]. Although the particle distribution was narrow, the toasting method used higher temperatures for a shorter period, suggesting that the increased rates of carbonization restricted the growth of the CQD particles. This was demonstrated by the comparatively smaller nanocrystals. On the other hand, the hydrothermal process provided a slower and longer heating profile, resulting in slightly larger nanoparticles. The average diameter difference between the two techniques was approximately 2 nm. 

All six samples showed excitation-dependent emission regardless of the fabrication technique, which is a typical optical property of CQDs [53,54]. The engrossment of multiple discrete electronic states due to the presence of different types of aggregates in ensemble fluorescence spectroscopy leads to excitation-dependent shifting of the emission spectra of CQDs. Further, surface-exposed functional groups play a crucial role in determining the extent and nature of aggregation and, thus, excitation-dependent or -independent emission spectra [55]. Even though the emission intensities of the samples from the hydrothermal method was higher than those from the toasting method, their fluorescence capabilities in cells are comparable.

The FTIR spectra of all of the samples closely resembled that of a parental carbohydrate molecule [56]. Since no additional chemicals were added during the two fabrication processes, the functional groups were related to hydrocarbons. The sample obtained from the hydrothermal technique contained significantly more -OH groups. The -OH functional group passivated the surface of the CQDs, increasing their emission and quantum yield [57,58,59]. According to the previous reports, the quantum yields of CQDs obtained from edible resources are generally low [41]. In this study, the CQDs obtained from bread also showed an up to 0.81% quantum yield; however, this was adequate for bioimaging cancer cells. The reason for the high quantum yield in the whole meal bread derived CQDs could be related to -OH surface groups and nitrogen doping. To prove this, soy flour was added to the precursor to increase the nitrogen content, and lemon juice was introduced to increase the surface functional groups. The quantum yields were improved to 1.42% and 2.32%, respectively. A detailed investigation on the underlying mechanism is ongoing.

### 4.3. Internalization of CQD

All six tested CQDs were able to cross the cell membrane barrier and readily internalized by both types of colon cancer cells tested, namely, CT-26 (mouse) and HT-29 (human), as shown by the confocal microscopy images. Similar results were obtained for CQDs from various sources using different fabrication techniques [60]. The cytotoxicity of the CQDs was negligible, making them biocompatible and suitable for several biological applications like bioimaging, biosensing, and drug targeting. The fluorescence images of cells obtained from various bread sources and fabrication methods were very similar. 

Rhodamine (RhB) dye is the common fluorescence labeling agent because of its excellent optical properties, such as high fluorescence quantum yield (65–97%), long excitation wavelengths, and high photo-stability. However, RhB dye even at a very low concentrations, could cause potential carcinogenicity and neurotoxicity. DAPI is another reagent with high quantum yield (~58%). However, the concentration of DAPI needed for live cell staining is generally very high; DAPI binds strongly to DNA and has mutagenicity and carcinogenic effects. In contrast, the CQDs prepared in this study are biocompatible. The natural origin and green synthesis route are added advantages to these CQDs. Henceforward, in vitro studies suggest that the CQDs prepared with simple and fast toasting methods can be excellent candidates for bioimaging and show potential applications for biosensing, targeted drug delivery, and disease diagnostics. 

## 5. Conclusions

In this work, CQDs were fabricated from three types of breads using toasting and hydrothermal routes. All six samples from both fabrication techniques were fluorescent in the absence of any additional surface modification techniques or doping materials. These CQDs were noncytotoxic and successfully used to image human colon cancer cells and mouse colon cancer cells. They were able to cross the nuclear membrane of the cell and remained in the cytoplasm to image. The toasting method is a faster, simpler, and cheaper route compared to the hydrothermal method. The toasting method consumed five times less energy than the hydrothermal method; however, it produced CQDs with comparable bioimaging capability. In addition, the toasting method does not require any specific apparatus, trained personnel, or chemicals. This efficient fabrication route could potentially be scaled up to commercial levels for other nanomedicine applications. The current CQDs prepared by toasting method have a low quantum yield. Our future work will include introducing some additional natural resources to the bread to enhance the quantum yield and understand the underlying mechanism.

## Figures and Tables

**Table 1:** Quantum yield measurements of all six samples.

Sample	QY (%)
CQD T1	0.03
CQD T2	0.20
CQD T3	0.05
CQD H1	0.33
CQD H2	0.81
CQD H3	0.63

**Table 2:** Comparison of the average energy consumptions between the toasting method and hydrothermal method.

Process	Toasting Method	Hydrothermal Method
Heating	249 KJ	23,040 KJ
Sonication	180 KJ	180 KJ
Centrifugation	84 KJ	84 KJ
Total energy consumed	513 KJ	2564 KJ

## Data Availability

Not applicable.

## References

[1] Boakye-Yiadom K.O., Kesse S., Opoku-Damoah Y., Filli M.S., Aquib M., Joelle M.M.B., Farooq M.A., Mavlyanova R., Raza F., Bavi R. (2019). Carbon dots: Applications in bioimaging and theranostics. Int. J. Pharm..

[2] Liu W., Li C., Ren Y., Sun X., Pan W., Li Y., Wang J., Wang W. (2016). Carbon dots: Surface engineering and applications. J. Mater. Chem. B.

[3] Singh R., Kumar R., Singh D., Savu R., Moshkalev S. (2019). Progress in microwave-assisted synthesis of quantum dots (graphene/carbon/semiconducting) for bioapplications: A review. Mater. Today Chem..

[4] Zheng X.T., Ananthanarayanan A., Luo K.Q., Chen P. (2015). Glowing graphene quantum dots and carbon dots: Properties, syntheses, and biological applications. Small.

[5] Ahmed S.R., Sherazee M., Srinivasan S., Rajabzadeh A.R. (2022). Nanozymatic detection of thiocyanate through accelerating the growth of ultra-small gold nanoparticles/graphene quantum dots hybrids. Food Chem..

[6] Xu X., Ray R., Gu Y., Ploehn H.J., Gearheart L., Raker K., Scrivens W.A. (2004). Electrophoretic analysis and purification of fluorescent single-walled carbon nanotube fragments. J. Am. Chem. Soc..

[7] Sun Y.-P., Zhou B., Lin Y., Wang W., Fernando K.S., Pathak P., Meziani M.J., Harruff B.A., Wang X., Wang H. (2006). Quantum-sized carbon dots for bright and colorful photoluminescence. J. Am. Chem. Soc..

[8] Xu B., Xu J., Zheng J., Yang Y., Liu X. (2019). Application advances of carbon quantum dots in optoelectronic devices. Chin. Sci. Bull..

[9] Yuan T., Meng T., He P., Shi Y., Li Y., Li X., Fan L., Yang S. (2019). Carbon quantum dots: An emerging material for optoelectronic applications. J. Mater. Chem. C.

[10] Li X., Rui M., Song J., Shen Z., Zeng H. (2015). Carbon and graphene quantum dots for optoelectronic and energy devices: A review. Adv. Funct. Mater..

[11] Qu Z., Liu L., Sun T., Hou J., Sun Y., Yu M., Diao Y., Lu S., Zhao W., Wang L. (2020). Synthesis of bifunctional carbon quantum dots for bioimaging and anti-inflammation. Nanotechnology.

[12] Jhonsi M.A. (2018). Carbon quantum dots for bioimaging. State of the Art in Nano-Bioimaging.

[13] Atchudan R., Edison T.N.J.I., Shanmugam M., Perumal S., Somanathan T., Lee Y.R. (2021). Sustainable synthesis of carbon quantum dots from banana peel waste using hydrothermal process for in vivo bioimaging. Phys. E Low-Dimens. Syst. Nanostruct..

[14] Wu F., Su H., Wang K., Wong W.-K., Zhu X. (2017). Facile synthesis of N-rich carbon quantum dots from porphyrins as efficient probes for bioimaging and biosensing in living cells. Int. J. Nanomed..

[15] Li Y., Bai G., Zeng S., Hao J. (2019). Theranostic carbon dots with innovative NIR-II emission for in vivo renal-excreted optical imaging and photothermal therapy. ACS Appl. Mater. Interfaces.

[16] Liu J., Li D., Zhang K., Yang M., Sun H., Yang B. (2018). One-step hydrothermal synthesis of nitrogen-doped conjugated carbonized polymer dots with 31% efficient red emission for in vivo imaging. Small.

[17] Khan M.E., Mohammad A., Yoon T. (2022). State-of-the-art developments in carbon quantum dots (CQDs): Photo-catalysis, bio-imaging, and bio-sensing applications. Chemosphere.

[18] Bhattacharya T., Shin G.H., Kim J.T. (2023). Carbon dots: Opportunities and challenges in cancer therapy. Pharmaceutics.

[19] Jia Q., Zhao Z., Liang K., Nan F., Li Y., Wang J., Ge J., Wang P. (2020). Recent advances and prospects of carbon dots in cancer nanotheranostics. Mater. Chem. Front..

[20] Shen C.-L., Liu H.-R., Lou Q., Wang F., Liu K.-K., Dong L., Shan C.-X. (2022). Recent progress of carbon dots in targeted bioimaging and cancer therapy. Theranostics.

[21] Sun X., Lei Y. (2017). Fluorescent carbon dots and their sensing applications. TrAC Trends Anal. Chem..

[22] Fan J., Kang L., Cheng X., Liu D., Zhang S. (2022). Biomass-Derived Carbon Dots and Their Sensing Applications. Nanomaterials.

[23] Shellaiah M., Sun K.W. (2023). Review on Carbon Dot-Based Fluorescent Detection of Biothiols. Biosensors.

[24] Caglayan M.O., Mindivan F., Şahin S. (2022). Sensor and bioimaging studies based on carbon quantum dots: The green chemistry approach. Crit. Rev. Anal. Chem..

[25] Yuan F., Li S., Fan Z., Meng X., Fan L., Yang S. (2016). Shining carbon dots: Synthesis and biomedical and optoelectronic applications. Nano Today.

[26] Liu M.L., Chen B.B., Li C.M., Huang C.Z. (2019). Carbon dots: Synthesis, formation mechanism, fluorescence origin and sensing applications. Green Chem..

[27] Anwar S., Ding H., Xu M., Hu X., Li Z., Wang J., Liu L., Jiang L., Wang D., Dong C. (2019). Recent advances in synthesis, optical properties, and biomedical applications of carbon dots. ACS Appl. Bio Mater..

[28] Namdari P., Negahdari B., Eatemadi A. (2017). Synthesis, properties and biomedical applications of carbon-based quantum dots: An updated review. Biomed. Pharmacother..

[29] Crista D., Esteves da Silva J.C., Pinto da Silva L. (2020). Evaluation of different bottom-up routes for the fabrication of carbon dots. Nanomaterials.

[30] Reza M.T. (2013). Upgrading Biomass by Hydrothermal and Chemical Conditioning.

[31] Berge N.D., Ro K.S., Mao J., Flora J.R., Chappell M.A., Bae S. (2011). Hydrothermal carbonization of municipal waste streams. Environ. Sci. Technol..

[32] Bandura A.V., Lvov S.N. (2006). The ionization constant of water over wide ranges of temperature and density. J. Phys. Chem. Ref. Data.

[33] Dong Y., Shao J., Chen C., Li H., Wang R., Chi Y., Lin X., Chen G. (2012). Blue luminescent graphene quantum dots and graphene oxide prepared by tuning the carbonization degree of citric acid. Carbon.

[34] Qi H., Teng M., Liu M., Liu S., Li J., Yu H., Teng C., Huang Z., Liu H., Shao Q. (2019). Biomass-derived nitrogen-doped carbon quantum dots: Highly selective fluorescent probe for detecting Fe3+ ions and tetracyclines. J. Colloid Interface Sci..

[35] Jin H., Gui R., Wang Y., Sun J. (2017). Carrot-derived carbon dots modified with polyethyleneimine and nile blue for ratiometric two-photon fluorescence turn-on sensing of sulfide anion in biological fluids. Talanta.

[36] Lai Z., Guo X., Cheng Z., Ruan G., Du F. (2020). Green synthesis of fluorescent carbon dots from cherry tomatoes for highly effective detection of trifluralin herbicide in soil samples. ChemistrySelect.

[37] Tyagi A., Tripathi K.M., Singh N., Choudhary S., Gupta R.K. (2016). Green synthesis of carbon quantum dots from lemon peel waste: Applications in sensing and photocatalysis. RSC Adv..

[38] Bandi R., Gangapuram B.R., Dadigala R., Eslavath R., Singh S.S., Guttena V. (2016). Facile and green synthesis of fluorescent carbon dots from onion waste and their potential applications as sensor and multicolour imaging agents. RSC Adv..

[39] Boruah A., Saikia M., Das T., Goswamee R.L., Saikia B.K. (2020). Blue-emitting fluorescent carbon quantum dots from waste biomass sources and their application in fluoride ion detection in water. J. Photochem. Photobiol. B Biol..

[40] Zhou J., Sheng Z., Han H., Zou M., Li C. (2012). Facile synthesis of fluorescent carbon dots using watermelon peel as a carbon source. Mater. Lett..

[41] Sk M.P., Jaiswal A., Paul A., Ghosh S.S., Chattopadhyay A. (2012). Presence of amorphous carbon nanoparticles in food caramels. Sci. Rep..

[42] Saxena M., Sarkar S. (2013). Fluorescence imaging of human erythrocytes by carbon nanoparticles isolated from food stuff and their fluorescence enhancement by blood plasma. Mater. Express.

[43] Eswaran S.G., Thiruppathi D., Vasimalai N. (2022). Synthesis of highly fluorescent carbon dots from bread waste and their nanomolar lead ions sensor application. Environ. Nanotechnol. Monit. Manag..

[44] Anpalagan K.A., Karakkat J.V., Truskewycz A., Saedi A.A., Joseph P., Apostolopoulos V., Nurgali K., Cole I., Cai Z., Lai D.T.H. (2020). Bioimaging of C2C12 Muscle Myoblasts Using Fluorescent Carbon Quantum Dots Synthesized from Bread. Nanomaterials.

[45] Kesberg A.I., Schleheck D. (2013). Improved protocol for recovery of bacterial DNA from water filters: Sonication and backflushing of commercial syringe filters. J. Microbiol. Methods.

[46] Fengel D. (1992). Characterization of cellulose by deconvoluting the OH valency range in FTIR spectra. Holzforschung.

[47] Ferreira-Villadiego J., Garcia-Echeverri J., Mejia M.V., Pasqualino J., Meza-Catellar P., Lambis H. (2018). Chemical modification and characterization of starch derived from plantain (*Musa paradisiaca*) peel waste, as a source of biodegradable material. Chem. Eng. Trans..

[48] Das P., Ganguly S., Margel S., Gedanken A. (2021). Tailor made magnetic nanolights: Fabrication to cancer theranostics applications. Nanoscale Adv..

[49] Lin L., Xia Y., Wen H., Lu W., Li Z., Xu H., Zhou J. (2023). Green and continuous microflow synthesis of fluorescent carbon quantum dots for bio-imaging application. AIChE J..

[50] Karakoc<monospace>̧</monospace>ak B.B.m., Laradji A., Primeau T., Berezin M.Y., Li S., Ravi N. (2020). Hyaluronan-conjugated carbon quantum dots for bioimaging use. ACS Appl. Mater. Interfaces.

[51] Khan Z.M., Rahman R.S., Islam S., Zulfequar M. (2019). Hydrothermal treatment of red lentils for the synthesis of fluorescent carbon quantum dots and its application for sensing Fe^3+^. Opt. Mater..

[52] Shen T., Wang Q., Guo Z., Kuang J., Cao W. (2018). Hydrothermal synthesis of carbon quantum dots using different precursors and their combination with TiO_2_ for enhanced photocatalytic activity. Ceram. Int..

[53] Sun Z., Li X., Wu Y., Wei C., Zeng H. (2018). Origin of green luminescence in carbon quantum dots: Specific emission bands originate from oxidized carbon groups. New J. Chem..

[54] Mintz K.J., Zhou Y., Leblanc R.M. (2019). Recent development of carbon quantum dots regarding their optical properties, photoluminescence mechanism, and core structure. Nanoscale.

[55] Sharma A., Gadly T., Gupta A., Ballal A., Ghosh S.K., Kumbhakar M. (2016). Origin of excitation dependent fluorescence in carbon nanodots. J. Phys. Chem. Lett..

[56] Wiercigroch E., Szafraniec E., Czamara K., Pacia M.Z., Majzner K., Kochan K., Kaczor A., Baranska M., Malek K. (2017). Raman and infrared spectroscopy of carbohydrates: A review. Spectrochim. Acta Part A Mol. Biomol. Spectrosc..

[57] Li M., Cushing S.K., Zhou X., Guo S., Wu N. (2012). Fingerprinting photoluminescence of functional groups in graphene oxide. J. Mater. Chem..

[58] Kubo T., Isobe T., Senna M. (2002). Enhancement of photoluminescence of ZnS: Mn nanocrystals modified by surfactants with phosphate or carboxyl groups via a reverse micelle method. J. Lumin..

[59] Liu P., Zhang C., Liu X., Cui P. (2016). Preparation of carbon quantum dots with a high quantum yield and the application in labeling bovine serum albumin. Appl. Surf. Sci..

[60] Azam N., Najabat Ali M., Javaid Khan T. (2021). Carbon Quantum Dots for Biomedical Applications: Review and Analysis. Front. Mater..

